# Comprehensive plastome variation and RNA editing in *Mentha*: insights into phylogenetic relationships and candidate DNA barcodes

**DOI:** 10.3389/fpls.2026.1889271

**Published:** 2026-08-18

**Authors:** Yujie He, Chengwen Gao

**Affiliations:** 1 Medical Research Center, The Affiliated Hospital of Qingdao University, Qingdao University, Qingdao, Shandong, China; 2 West Coast Second Hospital of Qingdao University Medical Group, Qingdao, Shandong, China

**Keywords:** comparative genomics, mentha, phylogeny, plastome, RNA editing

## Abstract

**Introduction:**

Mentha is an economically and medicinally important genus in Lamiaceae, but its taxonomy and species delimitation remain challenging because of frequent hybridization, polyploidy, and marked morphological plasticity.

**Methods:**

In this study, we comparatively analyzed 12 plastomes representing major Mentha species, hybrid taxa, and unresolved accessions, including four newly assembled genomes, to characterize plastome structure, repeat composition, sequence divergence, phylogenetic relationships, and plastid RNA editing. The *M. arvensis* plastome and RNA-seq datasets originated from independent Swiss and Indian accessions, respectively.

**Results:**

The plastomes were highly conserved in overall organization, ranging from 151,824 to 152,154 bp and displaying the typical quadripartite structure. Gene content and order were largely stable across taxa, with only minor variation likely associated with annotation differences at IR/SC boundary regions. Codon usage analysis revealed a clear bias toward A/U-ending synonymous codons, and most shared protein-coding genes showed low Ka/Ks ratios, indicating predominant purifying selection. Repeat analyses showed that simple sequence repeats were mainly composed of A/T-rich mononucleotide motifs, whereas long repeats were concentrated in the 30–40 bp size class. Comparative analyses identified six hypervariable regions, namely ccsA–ndhD, ycf1, ndhD, rpl32–trnL-UAG, rbcL–accD, and petA–psbJ, which represent promising candidate plastid markers for species discrimination. Phylogenetic analysis based on complete plastomes provided strong support for relationships among the sampled taxa and recovered a close affinity among *M. aquatica, M. arvensis,* and *M. canadensis*. In addition, RNA-seq analysis of *M. arvensis* identified 17 candidate plastid RNA editing sites, most of which were C-to-U conversions and nonsynonymous events.

**Discussion:**

Together, these results expand plastid genomic resources for Mentha and provide a useful framework for phylogenetic inference, species identification, and future germplasm utilization.

## Graphical Abstract

**Figure:**
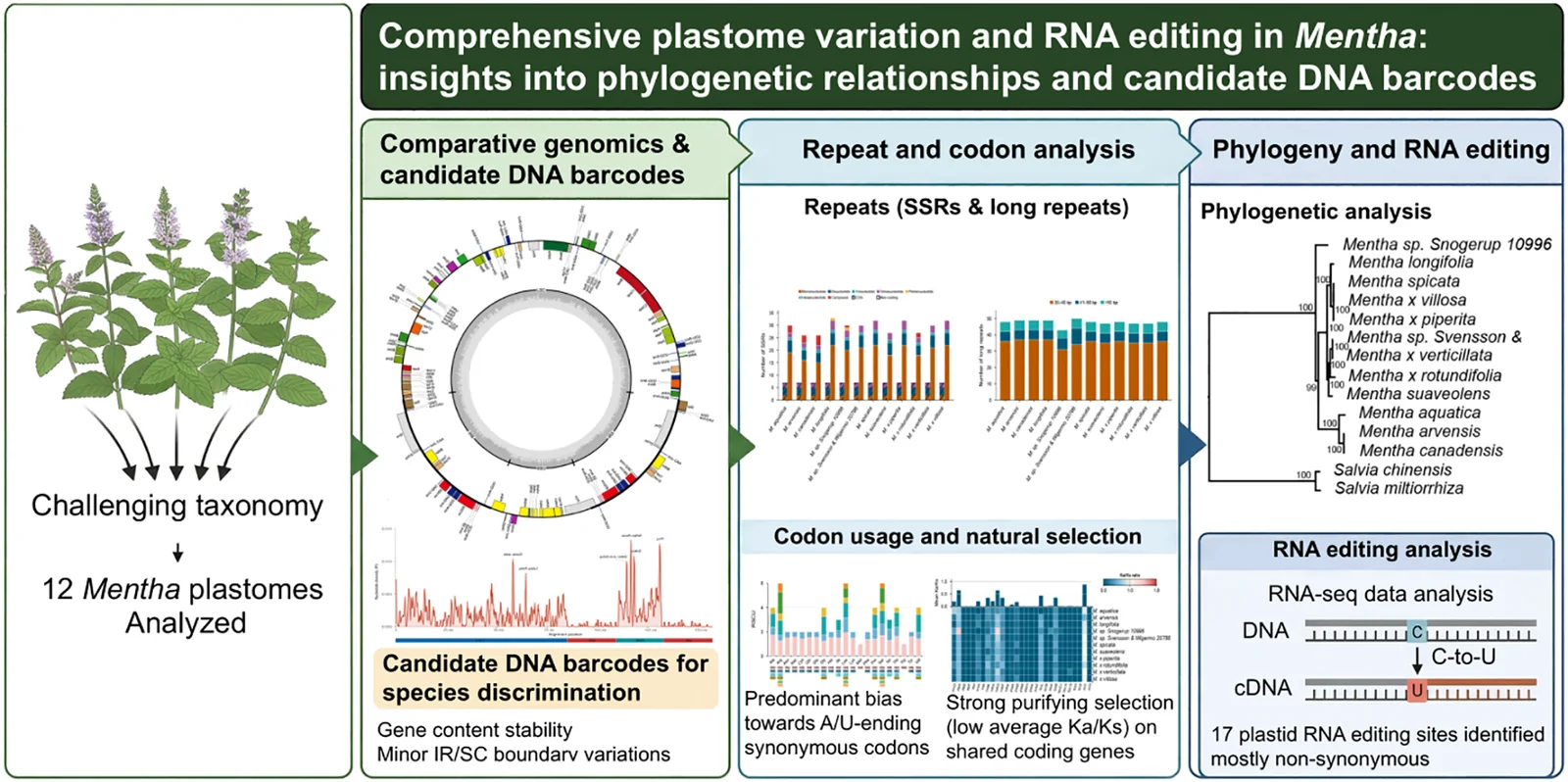

## 1 Introduction

The genus *Mentha* L. comprises aromatic perennial herbs of high medicinal, culinary, and industrial value and includes numerous cultivated species and natural hybrids (Salehi et al., 2018; Song et al., 2026). Different mint taxa are valued for their diverse terpene-rich chemotypes, including menthol-, carvone-, and pulegone-related profiles, which support extensive use in medicine, foods, cosmetics, and other commercial products (Yu et al., 2025; Song et al., 2026). Because economically important traits are often associated with particular species, hybrids, or cultivated lines, accurate identification of *Mentha* germplasm is essential for breeding, quality control, and resource management (Vining et al., 2020). However, species delimitation within *Mentha* remains problematic because morphological traits are highly plastic and are further complicated by frequent interspecific hybridization and polyploidy (Heylen et al., 2021; Tyler et al., 2025). As a result, accessions with similar morphology may not share the same evolutionary background, whereas hybrid derivatives may be maintained under stable horticultural or medicinal names (Tyler et al., 2025). In such a genus, taxonomic uncertainty is not only a systematic problem, but also a practical barrier to germplasm conservation, utilization, and authentication.

Plastid genomes provide a particularly useful framework for studying *Mentha* because they are structurally conserved, generally maternally inherited, and still contain enough sequence variation to inform species discrimination and phylogenetic inference (Rogalski et al., 2015). In a reticulate genus such as *Mentha*, plastome data are especially informative for tracing maternal lineages, identifying divergence hotspots, and developing chloroplast markers for difficult taxa (Tyler et al., 2025). Recent plastome studies of *M. aquatica* and *M. suaveolens* have shown that *Mentha* plastid genomes are broadly conserved, while still harboring hypervariable regions and useful phylogenetic signal (Magdy et al., 2025; Soorni and Golchini, 2025). However, existing work remains limited in taxon coverage, particularly for newly assembled accessions, hybrid materials, and unresolved *Mentha* samples. Besides DNA sequence variation, RNA editing adds a post-transcriptional layer of functional complexity to plant organelle genomes. In plastids, RNA editing most often involves C-to-U conversion and can restore conserved amino acids, alter protein properties, or modulate transcript function (Ichinose and Sugita, 2017; Small et al., 2020). Although plastid RNA editing has been characterized in many angiosperms, it is still poorly understood in *Mentha*. Integrating comparative plastome analysis with RNA editing information may therefore improve both evolutionary interpretation and the functional annotation of plastid genes in mint species.

In this study, four newly assembled *Mentha* plastomes were annotated and analyzed together with eight previously published plastomes. We also identified plastid RNA editing sites in *M. arvensis* using RNA-seq data. Our objectives were to: (1) characterize the overall plastome architecture of representative *Mentha* taxa; (2) identify repeat-rich and highly divergent loci with potential value as molecular markers; (3) evaluate plastome-based phylogenetic relationships across named species, hybrids, and unresolved accessions; and (4) describe the RNA editing profile of *M. arvensis*. The resulting dataset expands genomic resources for *Mentha* and provides a comparative basis for species identification, maternal lineage tracing, and future utilization of mint germplasm.

## 2 Materials and methods

### 2.1 Plant materials and plastome assembly

The *Mentha* accessions analyzed in this study included four newly assembled plastomes and eight previously published plastomes (**Supplementary Table 1**). The newly assembled accessions comprised *M. arvensis* (PX971240)*, M.* sp. *Snogerup 10996* (PX971241)*, M.* sp. *Svensson & Wigermo 20766* (PX971242), and *M. × rotundifolia* (PX971243). The *M. arvensis* plastome was reconstructed from leaf-derived DNA of an accession collected in Switzerland, whereas the RNA-seq dataset used to investigate plastid RNA editing was generated independently from waterlogged stem tissue of a separate M. arvensis accession collected in India. Thus, the plastome and transcriptome datasets represent geographically distinct accessions. The other three plastomes were reconstructed from dried herbarium material originating from Greece and Sweden. Detailed accession-level provenance, including voucher or source identifiers, geographic locality, collection information, taxonomic status, material source, and linked database records, is provided in **Supplementary Table 1**.

Accession-specific sequencing metadata are provided in **Supplementary Table 1**. DNA from the three herbarium accessions was extracted using the DNeasy Plant Mini Kit. Libraries were sequenced as PE150 reads on a NovaSeq 6000 or HiSeq 4000, whereas the Swiss *M. arvensis* WGS library was sequenced as PE101 reads on a HiSeq 2000. The independent RNA-seq dataset used for RNA-editing analysis was derived from waterlogged stem tissue of an Indian *M. arvensis* accession and sequenced on a NovaSeq 6000. Read yields and plastome coverage ranged from 5.53 to 37.25 million read pairs and from 85.15× to 2,812.37×, respectively.

Raw reads from the four newly assembled accessions were quality-filtered using Trimmomatic v0.38 to remove adapter sequences and low-quality bases (Bolger et al., 2014). High-quality reads were assembled using SPAdes v3.6.1 (Bankevich et al., 2012), and plastid-derived contigs were subsequently identified and aligned against the reference plastome of *M. longifolia* (GenBank accession ON124917) using Geneious v2025.0.2 for manual ordering and orientation (Kearse et al., 2012). Complete plastome sequences were reconstructed after manual inspection. Each of the four newly assembled plastomes was first annotated with CPGAVAS2 (Liu et al., 2012). The four sequences were then re-annotated using GeSeq with the reference chloroplast option and *M. longifolia* ON124917 as the closest-relative reference (Tillich et al., 2017). The CPGAVAS2/PX and GeSeq models were compared locus by locus, and discrepancies in gene/CDS boundaries, exon-intron junctions, start and stop codons, and IR/SC junctions were resolved manually against ON124917 and conserved plastid feature integrity. Redundant overlapping GeSeq models were removed. All 116 accession-specific comparisons, supporting evidence, and final decisions are reported in **Supplementary Table 3**. To ensure consistency in comparative analyses, the same annotation standard was applied across the entire dataset. Finally, circular plastome maps were generated using OGDRAW v1.3.1 (Greiner et al., 2019).

### 2.2 Codon usage bias analysis

Protein-coding sequences shared across the 12 *Mentha* plastomes were extracted and used for codon usage analysis. Relative synonymous codon usage (RSCU) values were calculated according to the method of Sharp and Li (Sharp and Li, 1986). Codons with RSCU values greater than 1 were regarded as preferentially used, whereas those with values lower than 1 were considered underrepresented relative to equal synonymous usage. This analysis was performed to characterize codon usage bias and to evaluate the degree of conservation in translational preference among *Mentha* plastomes. For the four newly assembled plastomes, RSCU calculations and visualization were additionally performed using the RSCU-Plot web application (https://pcg-lab.shinyapps.io/RSCU-Plot/) (Akrami et al., 2025; Diani Gohar and Soorni, 2025; Hejazi et al., 2025; Soorni and Golchini, 2025). The four accession-specific outputs were combined as **Supplementary Figure S2**.

### 2.3 Selective pressure analysis of shared protein-coding genes

Selective pressure on plastid coding genes was assessed using an ortholog-based strategy with *M. canadensis* as the reference plastome. All 79 shared protein-coding genes were extracted from the 12 *Mentha* plastomes and aligned using MAFFT v7.221 (Katoh and Standley, 2013). Ka, Ks, and Ka/Ks values were calculated with KaKs_Calculator using the MLWL model (Zhang, 2022). A Ka/Ks estimate was recorded as undefined when no or insufficient synonymous or nonsynonymous substitutions prevented a reliable ratio; this status did not indicate an absent gene. Genes with more than five undefined estimates across the 11 reference-versus-query comparisons were excluded, retaining 29 genes and excluding 50. For the retained genes, 54/319 undefined cells were replaced by the arithmetic mean of the estimable values for the same gene, and the completed matrix was used for the heatmap (**Supplementary Table 5A**). **Supplementary Table 5B** lists the excluded genes. Sensitivity summaries were additionally calculated from the 265 estimable raw values without imputation.

### 2.4 Identification of SSRs and long repeats

Simple sequence repeats (SSRs) were identified using MISA v2.1 (Beier et al., 2017). To distinguish genomic compartments, each of the 12 plastomes was partitioned by physical genomic coordinates. The coding pool comprised the union of all CDS exons, whereas the non-coding pool comprised intergenic spacers and introns; tRNA and rRNA exons and pseudogene-only spans were excluded. Both physical IR copies were retained, while overlapping annotations were counted once. Annotation coordinates were harmonized across sequence-identical records, and missing *ycf2* features in public records were restored only when donor and target intervals were sequence-identical. Within each plastome, disjoint segments were concatenated by compartment with 200-N separators to prevent SSR detection across artificial feature junctions; separator bases were excluded from compartment lengths. The minimum repeat thresholds were 10 for mononucleotides, 5 for dinucleotides, 4 for trinucleotides, and 3 for tetra-, penta-, and hexanucleotides, with a maximum interruption of 100 bp for compound SSRs. Abundance was summarized as both raw counts and SSRs per 10 kb of compartment sequence.

Long repeat sequences were identified using REPuter (Kurtz et al., 2001) with a minimum repeat size of 30 bp and a Hamming distance of 3. Forward and palindromic repeats were included in the analysis, and repeat abundance was summarized by length class. The repeat dataset was used to compare the extent of repeat-mediated variation across plastomes and to identify taxa with relatively higher repeat content.

### 2.5 IR boundary analysis and whole-plastome sequence comparison

Expansion and contraction of the IR regions were evaluated by comparing the junctions between IR and single-copy regions, namely JLB, JSB, JSA, and JLA, using IRscope (Amiryousefi et al., 2018). Particular attention was paid to genes located near or across junction boundaries, including *rps19*, *ycf1*, *ndhF*, *trnN-GUU*, and *rpl2*, in order to detect subtle structural variation among plastomes.

For global sequence comparison, plastome sequences were aligned using MAFFT v7.221 with default parameters (Katoh and Standley, 2013). The plastome of *M. canadensis* (NC_044082) was used as the reference for mVISTA analysis to visualize sequence conservation and divergence across the dataset (Frazer et al., 2004). Coding regions, introns, and intergenic spacers were compared simultaneously in order to determine whether divergence was concentrated in noncoding single-copy regions rather than in the IRs.

### 2.6 Nucleotide diversity and hotspot identification

Nucleotide diversity (π) across aligned plastomes was estimated with DnaSP v6.12 using a sliding window of 600 bp and a step size of 200 bp (Librado and Rozas, 2009). Regions with markedly elevated π values were designated as divergence hotspots and evaluated as candidate DNA barcodes for *Mentha*.

### 2.7 Phylogenetic analysis

Maximum likelihood (ML) phylogenetic analysis was performed using complete plastome sequences from all 12 *Mentha* accessions, with *Salvia chinensis* and *Salvia miltiorrhiza* designated as outgroups. Whole-genome alignments were generated with MAFFT v7.221 and inspected manually (Katoh and Standley, 2013). Tree inference was conducted in IQ-TREE v3.0.1 (Nguyen et al., 2015) under the best-fitting substitution model (TVM+F+I) identified by ModelFinder v3.7 (Kalyaanamoorthy et al., 2017). Node support was assessed using 1,000 ultrafast bootstrap replicates. To further evaluate the phylogenetic signal of individual hypervariable loci, separate ML trees were constructed for each of the six candidate DNA barcode regions identified by nucleotide diversity analysis.

### 2.8 Identification of plastid RNA editing sites

RNA editing sites in the *M. arvensis* plastome were identified from RNA-seq data. High-quality reads were mapped to the assembled plastome using HISAT2 v2.1.0 (Kim et al., 2019), and the resulting alignments were converted to BAM format with SAMtools v1.9 (Danecek et al., 2021). Variant calling was performed with BCFtools v1.9 (Danecek et al., 2021), and candidate editing sites were annotated and filtered using REDO v1.0 (Wu et al., 2018), which integrates editing type, codon position, and predicted amino acid change. To distinguish true RNA editing events from genomic SNPs, DNA-seq reads were independently mapped with Bowtie2 v2.3.5 (Langmead and Salzberg, 2012) and SNPs were called with BCFtools v1.9; sites with coincident DNA-level variation were excluded from the final dataset.

## 3 Results

### 3.1 General features of *Mentha* plastomes

**Figure 1:**
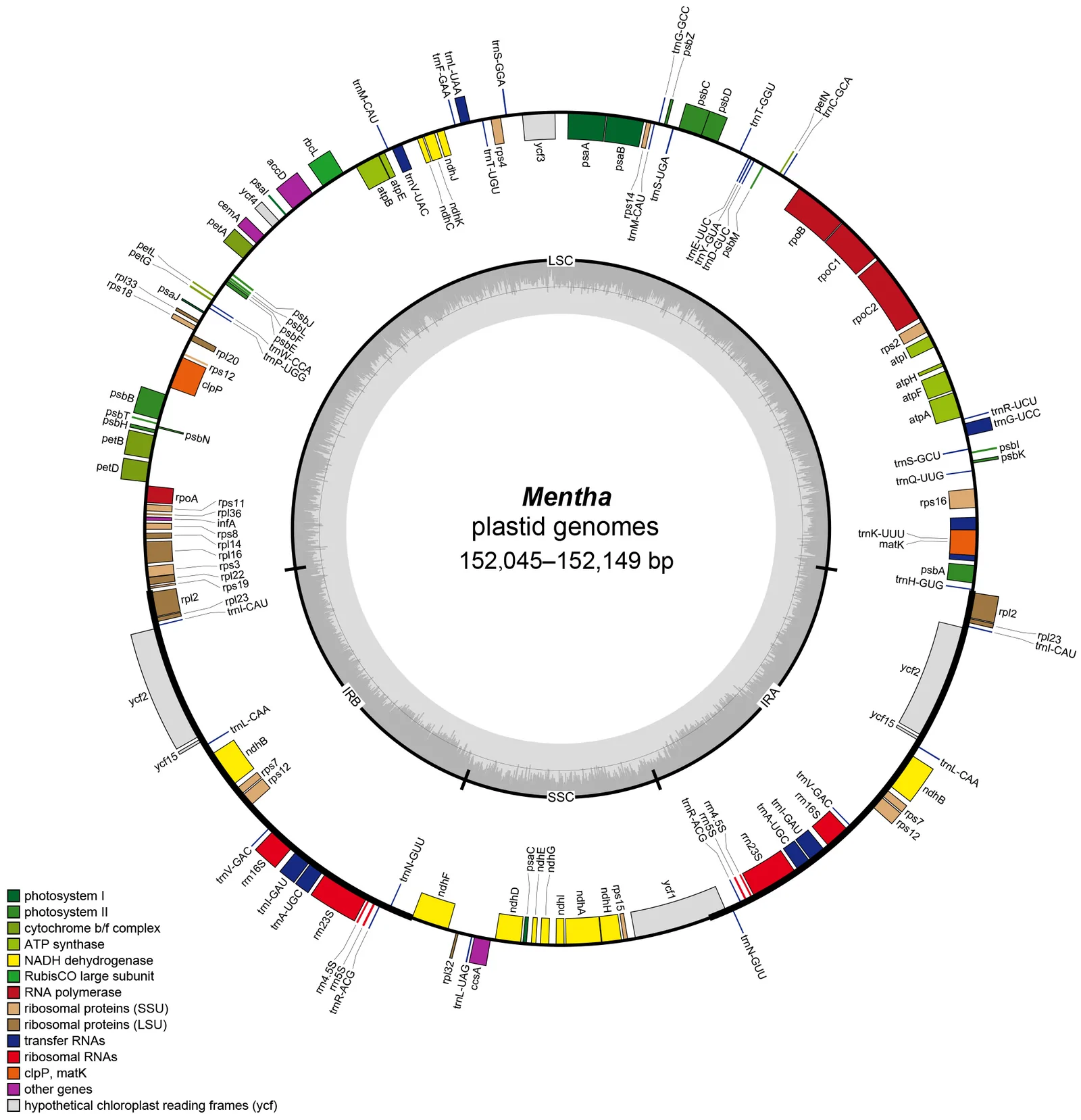
Circular gene map of representative *Mentha* plastid genomes. Genes on the inside are transcribed clockwise and those on the outside are transcribed counterclockwise. Functional categories are color coded.

In this study, four *Mentha* plastomes were newly assembled and annotated, expanding the comparative dataset to a total of 12 taxa. The 12 *Mentha* plastomes ranged from 151,824 bp (*M. suaveolens*) to 152,154 bp (*M. canadensis*), exhibiting the canonical quadripartite structure typical of angiosperm chloroplast genomes (**Figure 1**; **Table 1**). The LSC region spanned 82,962–83,279 bp, the SSC region 17,639–17,676 bp, and each of the two IR regions 25,600–25,625 bp. The overall GC content was uniform across taxa, ranging narrowly from 37.81% to 37.86%. Separate OGDRAW circular maps for the four newly assembled plastomes are provided in **Supplementary Figure S1**.

**Table 1:** Plastid genomic characteristics of *Mentha* species.

Species	Genome (bp)	LSC (bp)	IR (bp)	SSC (bp)	Number of total genes	Number of CDS	Number of tRNA genes	Number of rRNA genes	GC content (%)
*Mentha arvensis*	152149	83279	25606	17658	132	87	37	8	37.81
*Mentha* sp. *Snogerup 10996*	152131	83276	25608	17639	132	87	37	8	37.83
*Mentha* sp. *Svensson & Wigermo 20766*	152084	83170	25625	17664	132	87	37	8	37.85
*Mentha × rotundifolia*	152045	83172	25602	17669	132	87	37	8	37.86
*Mentha aquatica*	152077	83212	25600	17665	132	87	37	8	37.81
*Mentha canadensis*	152154	83278	25600	17676	132	87	37	8	37.81
*Mentha longifolia*	152078	83220	25603	17652	127	84	35	8	37.84
*Mentha spicata*	152048	83179	25608	17653	132	87	37	8	37.85
*Mentha suaveolens*	151824	82962	25602	17658	132	87	37	8	37.86
*Mentha × piperita*	152048	83179	25608	17653	132	87	37	8	37.85
*Mentha × verticillata*	152026	83165	25603	17655	131	86	37	8	37.85
*Mentha × villosa*	152048	83179	25608	17653	130	85	37	8	37.85

Gene content was largely conserved, with most plastomes encoding 132 genes comprising 87 protein-coding sequences (CDS), 37 transfer RNA (tRNA) genes, and 8 ribosomal RNA (rRNA) genes. *M. canadensis* and *M. × piperita* each encoded 132 genes (87 CDS, 37 tRNAs, and 8 rRNAs), whereas *M. × verticillata* encoded 131 genes (86 CDS, 37 tRNAs, and 8 rRNAs). *M. × villosa* and *M. longifolia* contained 130 and 127 genes, respectively, the latter with reduced CDS (84) and tRNA (35) complements (**Table 1**). These corrected totals are arithmetic sums of the reported feature classes. Functional annotation showed a typical angiosperm plastome gene set, with self-replication genes listed before photosynthesis-related genes in **Supplementary Table 2**. Intron-containing genes were conserved overall, with 23 genes harboring one or more introns; *clpP* and *ycf3* contained two introns, whereas *rps12* showed the expected trans-splicing structure. Several genes, including rRNAs and a subset of tRNAs and protein-coding genes, were duplicated in the IR regions.

### 3.2 Codon usage bias and selective constraint

**Figure 2:**
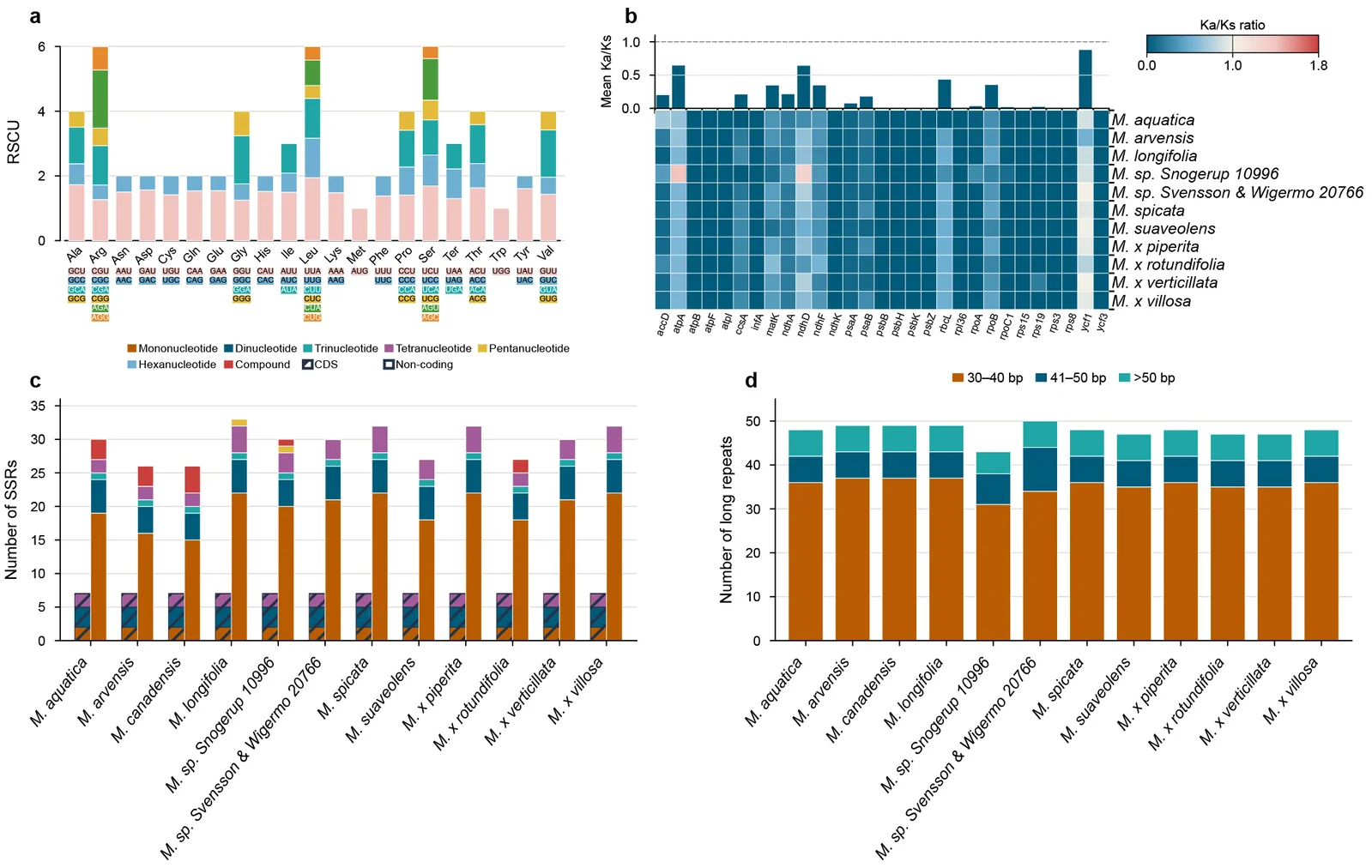
Molecular evolutionary features of *Mentha* plastomes. **(a)** Stacked bar chart of relative synonymous codon usage (RSCU) for each amino acid across 12 taxa. **(b)** Heatmap of gene-wise mean-imputed Ka/Ks ratios for 29 retained shared protein-coding genes. **(c)** Paired stacked bars of compartment-specific SSRs in CDS (hatched) and non-coding regions (intergenic spacers plus introns; solid), categorized by motif class and including compound SSRs. **(d)** Number of long repeats (≥ 30 bp) classified by length class per plastome.

RSCU analysis of protein-coding sequences shared across all 12 plastomes revealed a strong and consistent preference for synonymous codons ending in adenine (A) or uracil (U), in line with the AT-rich composition of angiosperm plastid genomes (**Figure 2a**; **Supplementary Table 4**). The five codons with the highest mean RSCU values across taxa were UUA (Leu, 1.95), AGA (Arg, 1.80), GCU (Ala, 1.73), UCU (Ser, 1.69), and ACU (Thr, 1.63). Methionine (AUG) and tryptophan (UGG), each encoded by a single codon, had RSCU values of 1.0. RSCU profiles were highly similar across the 12 taxa, indicating conserved plastid codon preferences irrespective of species boundaries or hybrid origin. Independent RSCU-Plot results for the four newly assembled plastomes reproduced the same A/U-ending preference and are shown in **Supplementary Figure S2**.

Ka/Ks ratios were estimable for 29 of the 79 shared protein-coding genes across 11 pairwise comparisons with *M. canadensis* (**Figure 2b**; **Supplementary Table 5A**). Of the 319 possible gene-by-comparison estimates, 265 were estimable, whereas 54 were undefined because there were insufficient synonymous and/or nonsynonymous substitutions for reliable ratio calculation. Undefined values were retained as missing and excluded from numerical summaries. Among the 265 estimable values, 261 (98.5%) were below 1, supporting predominant purifying selection among the evaluable comparisons. Four values exceeded 1: *atpA* in *M.* sp. Snogerup 10996 (1.396), *ndhD* in the same accession (1.258), and *ycf1* in *M.* sp. Svensson & Wigermo 20766 and *M. × verticillata* (1.008 each). Because these were isolated pairwise estimates and two were only marginally above 1, they were interpreted cautiously and were not considered evidence of positive selection. The 50 genes with more than five undefined estimates are listed in **Supplementary Table 5B**.

### 3.3 Simple sequence repeats and long repeats

The historical whole-plastome screen recovered 30–40 pure SSRs per plastome when compound loci were excluded (**Supplementary Table 6**), including 30 in *M. canadensis*, 31 in *M. arvensis*, and 32 in *M. × rotundifolia*. The compartment-specific rescan used a different counting definition: it included compound loci but excluded SSRs crossing CDS/non-coding boundaries, yielding 439 loci and 33–40 SSRs per plastome (**Figure 2c**; **Supplementary Table 7**). Of these loci, 84 occurred in CDS (seven per plastome) and 355 in non-coding regions (26–33 per plastome), including 299 intergenic and 56 intronic loci. Thus, non-coding regions contained 4.2-fold more SSRs than CDS and had a 5.5-fold higher length-normalized density (4.867 versus 0.885 SSRs per 10 kb). The coding pool was dominated by dinucleotide repeats (36; 42.9%), followed by mononucleotide and tetranucleotide repeats (24 each; 28.6%). Non-coding SSRs were dominated by mononucleotide repeats (236; 66.5%), followed by dinucleotide (56; 15.8%) and tetranucleotide repeats (36; 10.1%); trinucleotide, pentanucleotide, and compound SSRs accounted for 12, 2, and 13 loci, respectively. Five historical whole-plastome mononucleotide calls crossed a CDS/non-coding boundary and were therefore excluded from the compartment-specific totals.

Long dispersed repeats (≥ 30 bp) were detected across all 12 *Mentha* plastomes, with each accession harboring a comparable number of repeat elements (**Figure 2d**; **Supplementary Table 8**). The most abundant size class was 30–40 bp in all taxa, and repeats longer than 50 bp were present but comparatively scarce. Palindromic and forward repeats occurred in broadly comparable numbers. Overall, the total number of long repeats was relatively consistent across taxa, with no single accession showing a striking excess of long dispersed elements.

### 3.4 IR boundary comparison and whole-genome sequence conservation

**Figure 3:**
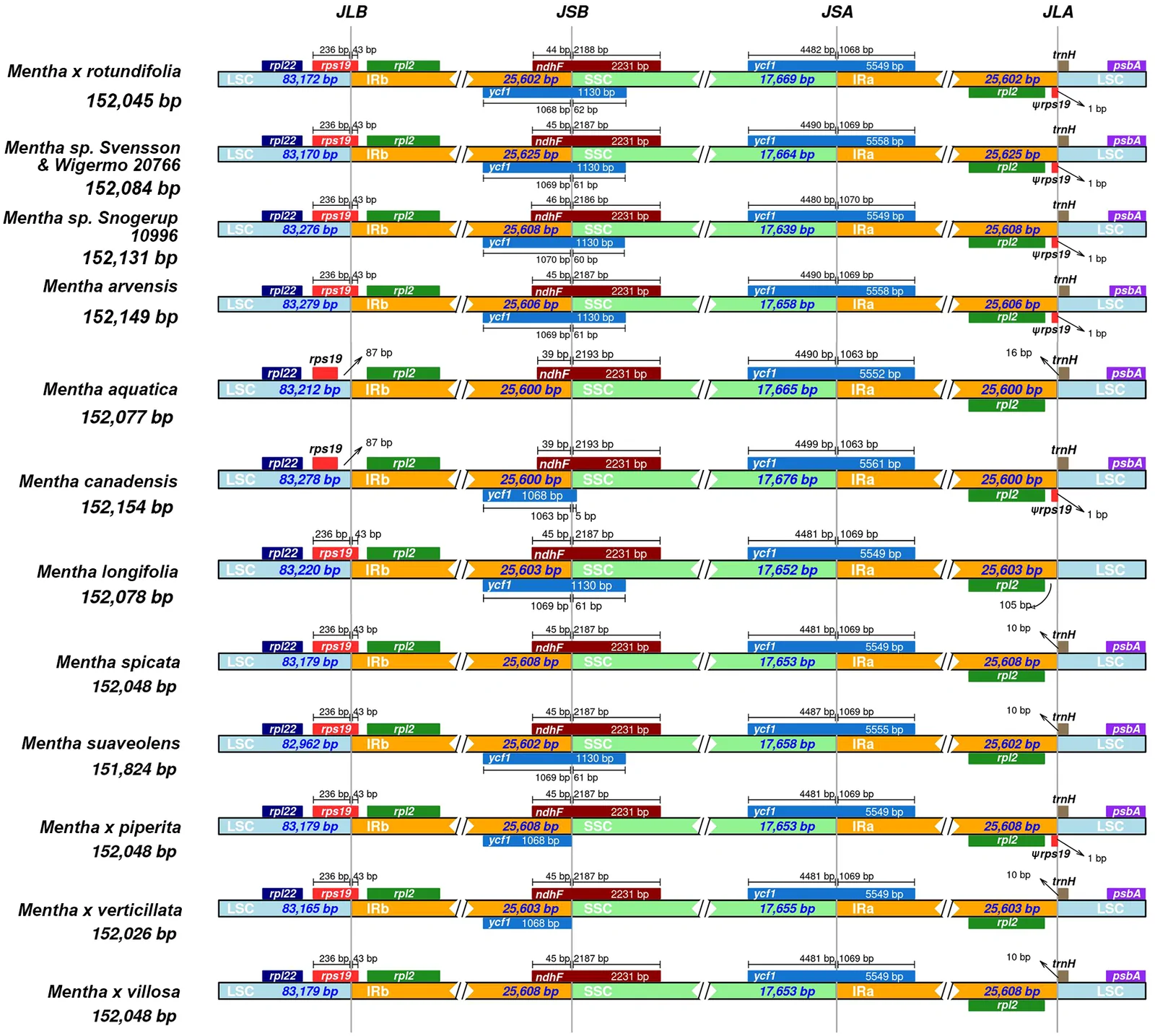
Comparison of IR/SC boundary structure among representative *Mentha* taxa. Junctions JLB, JSB, JSA, and JLA indicate LSC/IRb, IRb/SSC, SSC/IRa, and IRa/LSC boundaries, respectively. Gene positions and distances from the junctions are indicated in base pairs. The IRa-derived truncated copy of *rps19* is labeled ψrps19 in all taxa and is not counted as an intact protein-coding gene.

Comparison of IR/SC junction regions across the 12 *Mentha* plastomes revealed broadly conserved configurations with minor taxon-specific variation (**Figure 3**). At JLB, *rpl22* was located entirely within the LSC in all taxa. In *M. aquatica* and *M. canadensis*, *rps19* was confined to the LSC and located 87 bp from JLB. In the remaining ten accessions, *rps19* crossed JLB, with 236 bp located in the LSC and 43 bp extending into IRb. At JSB, *ndhF* extended 39–46 bp into IRb in all plastomes. A short IRb-derived ψycf1 copy was present at JSB in nine accessions and was absent from *M. aquatica*, *M. spicata*, and *M. × villosa*. The full-length *ycf1* gene crossed JSA in every taxon, with a 1,063–1,070 bp terminal segment located in IRa. At JLA, *trnH-GUG* was absent from *M. longifolia*. It directly abutted JLA in *M. × rotundifolia*, *M.* sp. *Svensson & Wigermo 20766, M.* sp. *Snogerup 10996, M. arvensis, M. canadensis*, and *M. × piperita*, whereas it was located 16 bp from JLA in *M. aquatica* and 10 bp from JLA in *M. spicata, M. suaveolens, M. × verticillata*, and *M. × villosa*.

**Figure 4:**
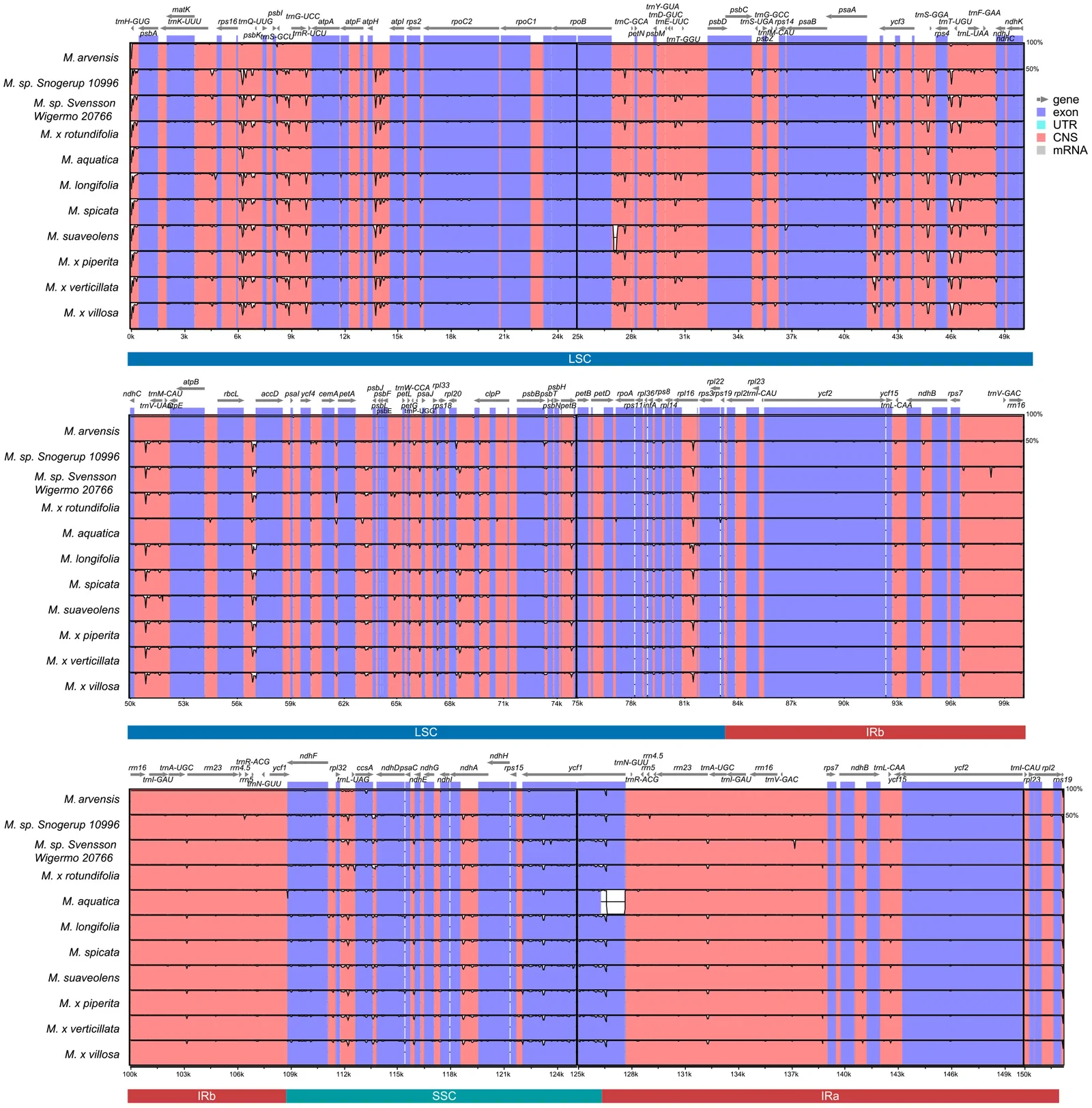
mVISTA visualization of 12 *Mentha* plastomes aligned against *M. canadensis* (NC_044082) as the reference. The horizontal axis represents genome position, and the vertical axis shows sequence identity (50–100%). The feature key identifies exons, untranslated regions (UTRs), conserved non-coding sequences (CNSs), and mRNAs. Regions of lower conservation appear as valleys in the identity tracks.

mVISTA alignment of the 12 plastomes against *M. canadensis* confirmed high overall sequence conservation (**Figure 4**). The IRs were generally more conserved than the single-copy regions, but they were not identical and retained visible taxon-specific differences. Greater divergence was concentrated in the LSC and SSC, particularly in intergenic spacers, including *trnH-GUG–psbA, rps16–trnQ-UUG, rbcL–accD, petA–psbJ, rpl32–trnL-UAG*, and *ccsA–ndhD*, whereas protein-coding sequences were comparatively conserved.

### 3.5 Nucleotide diversity and candidate barcoding loci

**Figure 5:**
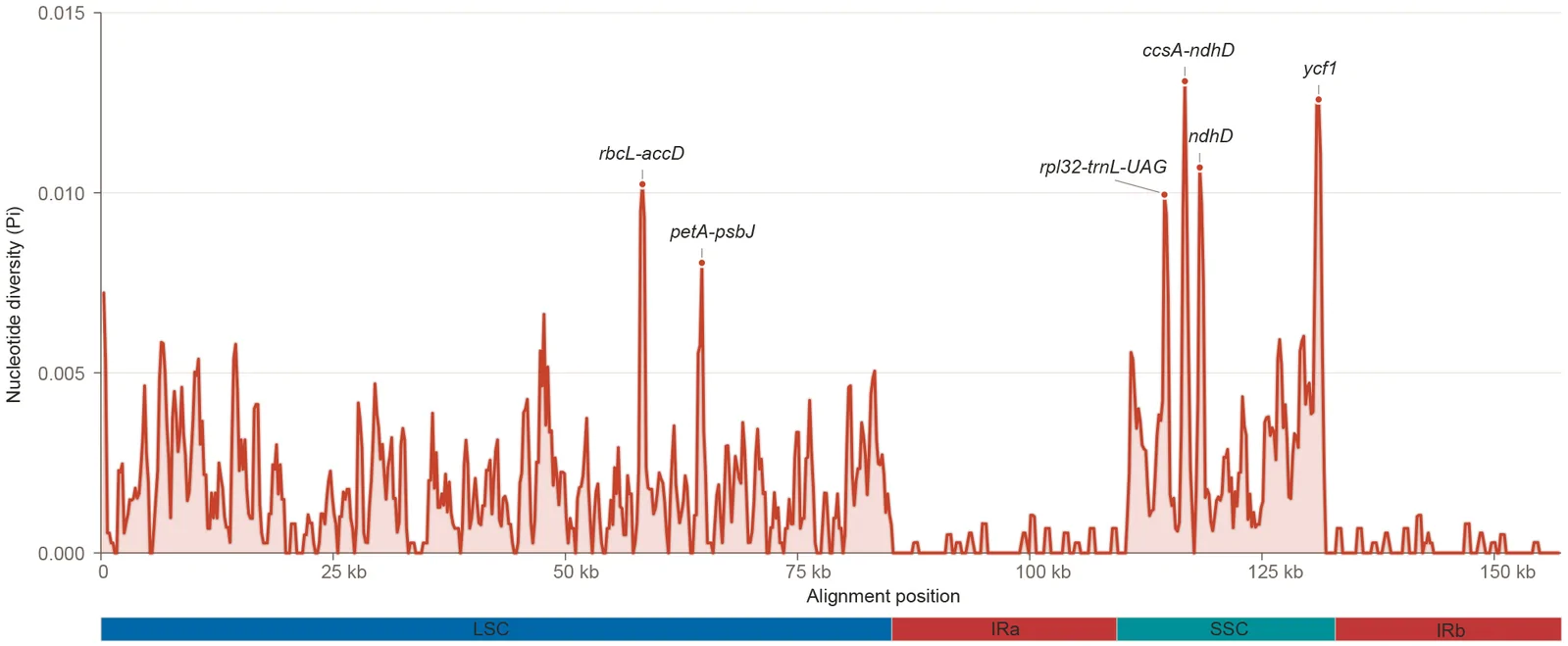
Sliding-window analysis of nucleotide diversity across aligned *Mentha* plastomes.

Sliding window analysis of nucleotide diversity across aligned Mentha plastomes identified six hypervariable regions: *ccsA–ndhD, ycf1, ndhD, rpl32–trnL-UAG, rbcL–accD*, and *petA–psbJ* (**Figure 5**). As anticipated, the IR regions exhibited substantially lower nucleotide diversity relative to the LSC and SSC, and the majority of peaks were confined to intergenic spacers and the *ycf1* coding region. These six hypervariable loci were selected as candidate DNA barcodes and evaluated individually through separate phylogenetic analysis.

### 3.6 Phylogenetic relationships

**Figure 6:**
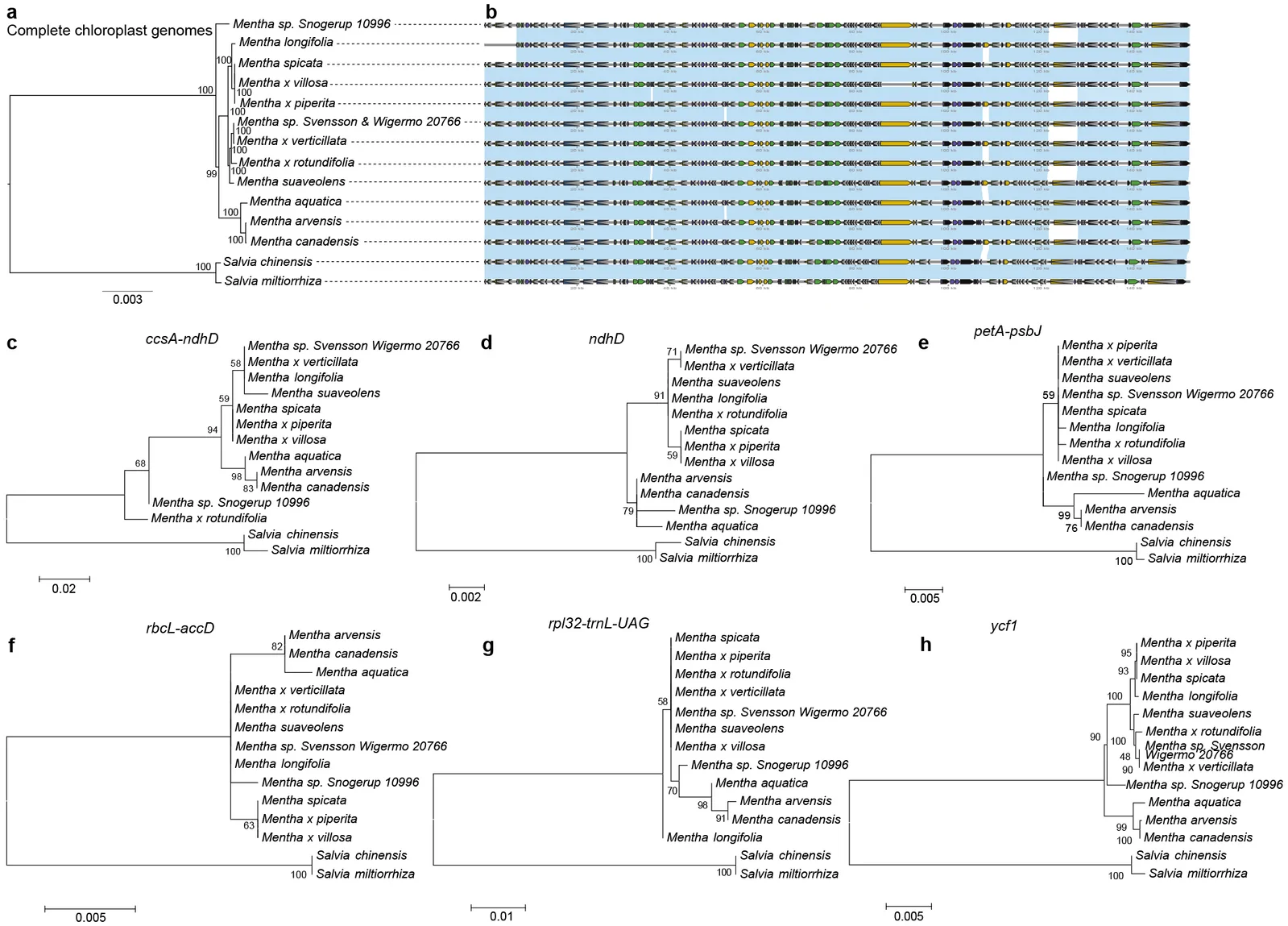
Phylogenetic trees of *Mentha* based on complete plastomes and individual candidate barcode regions. **(a)** Maximum likelihood tree based on complete chloroplast genomes. **(b)** Comparison panel showing aligned genomes. **(c–h)** Individual ML trees constructed from the six hypervariable loci: **(c)** *ccsA–ndhD*; **(d)** *ndhD*; **(e)** *petA–psbJ*; **(f)** *rbcL–accD*; **(g)** *rpl32–trnL-UAG*; **(h)** *ycf1*. *Salvia chinensis* and *Salvia miltiorrhiza* were used as outgroups throughout. Bootstrap support values are shown at nodes; scale bars indicate substitutions per site.

Maximum likelihood analysis of the 12 *Mentha* plastomes recovered a well-resolved and strongly supported topology, with *Mentha* strongly supported as monophyletic (**Figures 6a, b**). *M.* sp. *Snogerup 10996* was sister to the remaining taxa, which were divided into two main clades: one containing *M. aquatica, M. arvensis*, and *M. canadensis*, and the other including the remaining eight taxa. Overall, the plastome tree provided high phylogenetic resolution among the sampled *Mentha* taxa, although nuclear evidence will still be needed to clarify reticulate evolution within the genus.

Single-locus trees derived from the six candidate marker regions recovered broadly concordant topologies with the whole-plastome tree, though with reduced bootstrap support at some internal nodes (**Figures 6c–h**). *ycf1* provided the highest resolution among individual markers, recovering most major groupings with support values ≥ 90%. Minor topological discordances were observed in the placement of *M.* sp. *Snogerup 10996* and *M.* sp. *Svensson & Wigermo 20766* across markers, which may reflect the reticulate evolutionary history of these specimens or limited phylogenetic signal in single short loci.

### 3.7 RNA editing sites in the *Mentha arvensis* plastome

RNA editing analysis of the *Mentha arvensis* plastome identified 17 high-confidence editing sites distributed across 11 protein-coding genes and one tRNA gene (**Table 2**). Fourteen of the 17 events were C-to-U conversions, representing the predominant type of plastid RNA editing, whereas the remaining three comprised one A-to-G conversion in *ndhH*, one C-to-A transversion in *rbcL*, and one U-to-C synonymous substitution in *petB*. The *trnN-GUU* site occurred in a non-protein-coding context. Excluding this tRNA event, 15 of the 16 coding-region edits were nonsynonymous. The most frequent amino acid conversion was serine to leucine, detected in *atpI, psbZ, rps14*, and *ndhB*. Other recurrent changes included serine to phenylalanine in *matK* and *rpoA*, and proline to leucine in *atpF, petB*, and *ndhE*. Editing efficiency ranged from 13.7% at the *rbcL* site to 100%, with most sites showing relatively high editing levels. *matK* harbored the largest number of editing sites (four), followed by *petB* (three). Among the 16 coding-associated events, edits occurred mainly at the second codon position (11 sites), followed by the first position (4 sites) and the third position (1 site).

**Table 2:** RNA editing sites in the plastid genome of *Mentha arvensis* identified using RNA-sequencing data.

Gene name	Editing position in genome	Editing position in gene	Editing position in codon	Editing type	Codon change	Amino acid change	Coverage depth	Editing efficiency
*matK*	3399	155	2	C->U	UCC->UUC	S->F	8	87.5%
*matK*	2838	716	2	C->U	UCU->UUU	S->F	5	100.0%
*matK*	2374	1180	1	C->U	CGG->UGG	R->W	9	100.0%
*matK*	2305	1249	1	C->U	CAU->UAU	H->Y	9	88.9%
*atpF*	12957	92	2	C->U	CCA->CUA	P->L	8	75.0%
*atpI*	14668	629	2	C->U	UCA->UUA	S->L	6	100.0%
*psbZ*	35458	50	2	C->U	UCA->UUA	S->L	9	77.8%
*rps14*	36550	80	2	C->U	UCA->UUA	S->L	14	100.0%
*rbcL*	55594	673	1	C->A	CUU->AUU	L->I	51	13.7%
*petB*	74973	24	3	U->C	UUU->UUC	F->F	9	100.0%
*petB*	75367	418	1	C->U	CGG->UGG	R->W	10	100.0%
*petB*	75560	611	2	C->U	CCA->CUA	P->L	34	97.1%
*rpoA*	77962	200	2	C->U	UCC->UUC	S->F	10	100.0%
*trnN-GUU*	107485	3	–	C->U	–	–	18	100.0%
*ndhE*	116035	233	2	C->U	CCG->CUG	P->L	6	100.0%
*ndhH*	120395	881	2	A->G	CAC->CGC	H->R	5	100.0%
*ndhB*	139835	149	2	C->U	UCA->UUA	S->L	15	80.0%

## 4 Discussion

The comparative analysis of 12 *Mentha* plastomes presented here broadens the plastid genomic resources currently available for this genus and provides a quantitative framework for evaluating genome structure, sequence divergence, repeat composition, phylogenetic utility, and post-transcriptional modification. The *Mentha* plastomes were consistent with the conserved plastome architecture commonly observed in Lamiaceae and angiosperms (Wicke et al., 2011; Daniell et al., 2016; Soorni and Golchini, 2025; Gao et al., 2026). Although the sampled taxa include both recognized species and hybrid accessions, plastome organization remained highly conserved, suggesting that reticulate evolution in *Mentha* has not been accompanied by major shifts in overall plastid genome structure. The limited variation in total gene number among taxa is therefore more likely to reflect annotation differences associated with IR/SC boundary genes, especially *rps19* and *ycf1*, than substantial lineage-specific gene gain or loss.

The codon usage analysis showed a pronounced bias toward A/U-ending synonymous codons in all 12 plastomes. This pattern agrees well with the AT-rich compositional bias typical of plastid genomes and with the broader theoretical framework proposed by Sharp and Li (Sharp and Li, 1986), in which codon usage is shaped by the combined effects of mutation, drift, and selection. The high similarity of RSCU profiles across both species and hybrids indicates that codon preference in *Mentha* plastomes is evolutionarily stable and is unlikely to represent recent lineage-specific adaptation. This interpretation is further supported by the generally low Ka/Ks ratios observed for most shared coding genes, which indicate that plastid protein evolution in *Mentha* is dominated by purifying selection, as is typical for genes involved in photosynthesis, transcription, and translation in land-plant plastomes (Wicke et al., 2011; Geng et al., 2026). The comparatively elevated ratios detected for *ycf1* and a few *ndh*-associated loci may instead reflect localized relaxation of selective constraint or differential evolutionary rates rather than strong adaptive divergence.

Repeat analysis revealed that mononucleotide A/T-rich SSRs are the dominant repeat class in *Mentha*, whereas long repeats are concentrated mainly in the 30–40 bp size class. These features are consistent with the repeat profiles reported in recently published *Mentha* plastomes and more broadly with the AT-rich nature of plastid intergenic regions in angiosperms (Magdy et al., 2025; Soorni and Golchini, 2025). The compartment-resolved analysis showed that this pattern was driven mainly by non-coding sequence: intergenic spacers and introns contained 355 SSRs and an SSR density approximately 5.5 times that in CDS. Coding regions contained only seven SSR loci per plastome and displayed a different motif profile, with dinucleotide repeats forming the largest class. This contrast is consistent with stronger constraint on length-variable repeats in protein-coding sequence, whereas repeats in intergenic spacers and introns may tolerate greater length variation. The non-coding A/T- and AT/TA-rich loci are therefore the more appropriate candidates for cultivar authentication, germplasm evaluation, and population-level marker development. However, in silico abundance does not demonstrate polymorphism, and marker utility should be validated in population-level samples. The broadly conserved compartment-specific profiles also indicate that repeat-associated mutational processes have not produced major structural instability in the sampled *Mentha* plastomes.

The IR/SC boundary comparisons further support the conclusion that plastome evolution in *Mentha* is conservative but not static. Most taxa shared similar junction structures, whereas minor differences were concentrated in *rps19*, *ycf1*, *ndhF*, and adjacent regions. Such subtle IR expansions and contractions are a common source of small plastome-length differences in angiosperms and are typically associated with partial duplication or truncation of boundary genes rather than with large-scale rearrangements (Zhu et al., 2016; Cao et al., 2024). In the present study, the recurrent involvement of *ycf1* at the SSC/IR junction is especially informative because this gene is not only structurally dynamic but also one of the most sequence-variable loci in the plastome. The nucleotide diversity analysis identified six hypervariable regions, namely *ccsA–ndhD*, *ycf1*, *ndhD*, *rpl32–trnL-UAG*, *rbcL–accD*, and *petA–psbJ*, all of which showed phylogenetically informative variation. Their distribution mainly in the single-copy regions, rather than in the IRs, is consistent with the lower substitution rate generally associated with duplicated IR sequences (Daniell et al., 2016; Wu et al., 2021). Among these hotspots, *ycf1* showed the highest individual resolution, which agrees with the growing consensus that *ycf1* is among the most powerful plastid barcode loci for land plants (Dong et al., 2015). Collectively, these loci should therefore be regarded as candidate DNA barcodes for *Mentha*, with *ycf1* as the strongest single-locus candidate and the remaining regions as useful complementary markers in multilocus identification schemes.

The plastome phylogeny strongly supported the monophyly of *Mentha* and recovered a well-resolved internal topology. *M.* sp. *Snogerup 10996* was placed as the earliest-diverging lineage, whereas the remaining taxa largely formed two major clades, including one clade comprising *M. aquatica, M. arvensis*, and *M. canadensis*, and a second clade containing the remaining sampled taxa. The close relationship between *M. aquatica* and *M. canadensis* agrees with previous chloroplast-based evidence (Soorni and Golchini, 2025). Nevertheless, because plastomes primarily trace maternal inheritance, the present tree should be interpreted as a plastid-based evolutionary framework; nuclear data will still be required to resolve reticulate evolution in *Mentha* (Heylen et al., 2021).

The 17 plastid RNA editing sites identified in *M. arvensis* add a functional dimension to plastome evolution in *Mentha*. The predominance of C-to-U conversion is fully consistent with the canonical editing pattern described for land-plant chloroplasts (Ichinose and Sugita, 2017; Small et al., 2020). Most of the editing events in the present study were nonsynonymous, and the recurrent S→L and P→L substitutions are also in line with the widely recognized role of plastid RNA editing in restoring or reinforcing hydrophobic amino acid residues at functionally constrained sites (Yura and Go, 2008). The presence of a synonymous edit in *petB* and a site in *trnN-GUU* indicates that editing in *M. arvensis* is not restricted strictly to obvious protein-altering events. Moreover, the A-to-G event in *ndhH* and the C-to-A event in *rbcL* are unusual relative to the dominant plastid editing pattern and therefore deserve cautious interpretation until validated independently. The low editing efficiency observed at the *rbcL* site further suggests that some edits may be incomplete or condition-dependent rather than constitutive. Overall, the editing profile identified here is broadly compatible with the range of plastid RNA editing reported in other angiosperms and supports the view that post-transcriptional modification remains an important component of chloroplast gene regulation (Adamiec and Luciński, 2024; Ma et al., 2025).

This study also has limitations. First, the taxon sampling was informative but remains limited relative to the full diversity of *Mentha* and its many hybrid derivatives. Second, the plastid-based phylogeny traces only the maternal lineage and may not fully reflect reticulate evolution in a hybrid-rich genus. Third, RNA editing was analyzed only in *M. arvensis* because comparable plastid RNA-seq datasets were unavailable for PX971241–PX971243; editing in the other three newly assembled plastomes therefore could not be evaluated without new transcriptome data. Future matched DNA and RNA sampling will be required for valid cross-species comparisons. Even so, the present data support plastome-scale analyses for species identification and evolutionary interpretation in *Mentha*.

## 5 Conclusion

Comparative analysis of 12 *Mentha* plastid genomes showed that the genus possesses a highly conserved plastome structure but retains informative variation in repeat composition, IR boundary configuration, sequence-divergence hotspots, and plastid RNA editing. Codon-usage profiles were also strongly conserved, with all 12 plastomes preferentially using A/U-ending synonymous codons. The complete- plastome phylogeny strongly supported the monophyly of *Mentha* and clarified several internal relationships, while hotspot analysis highlighted *ycf1* and several additional loci as candidate DNA barcodes. The RNA-editing profile of *M. arvensis* further indicates that plastid post-transcriptional regulation represents another source of functional diversity. These results provide a genomic framework for future work on *Mentha* taxonomy, phylogeny, germplasm identification, and molecular breeding.

## Data Availability

The datasets presented in this study can be found in online repositories. The names of the repository/repositories and accession number(s) can be found in the article/**Supplementary Material**.

## Supplementary material

Supplementary Figure 1 Circular plastome maps of the four newly assembled *Mentha* plastomes: *M. arvensis* (PX971240), *M.* sp. *Snogerup 10996* (PX971241), *M. × rotundifolia* (PX971243), and *M.* sp. *Svensson & Wigermo 20766* (PX971242). Genes outside and inside each circle are transcribed counterclockwise and clockwise, respectively. Functional categories are color coded. The inner ring marks the LSC, SSC, IRa, and IRb regions; the positions of rps19, ycf1, ndhF, and trnH-GUG are shown at or near the IR/SC junctions.

Supplementary Figure 2 RSCU profiles of the four newly assembled *Mentha* plastomes generated with the RSCU-Plot web application, shown from top to bottom: *M. arvensis* (PX971240), *M.* sp. *Snogerup 10996* (PX971241), *M.* sp. *Svensson & Wigermo 20766* (PX971242), and *M. × rotundifolia* (PX971243). Bars show RSCU values for synonymous codons grouped by amino acid. Values greater than 1 indicate preferential codon use, whereas values below 1 indicate underrepresentation.
