# Supplementary material for: Comprehensive plastome variation and RNA editing in Mentha: insights into phylogenetic relationships and candidate DNA barcodes
Source: Front Plant Sci. 2026 Aug 18;17:1889271. doi: 10.3389/fpls.2026.1889271 (PMC13529761; doi:10.3389/fpls.2026.1889271)
Supplement: Supplementary file 1 [file DataSheet1.pdf]

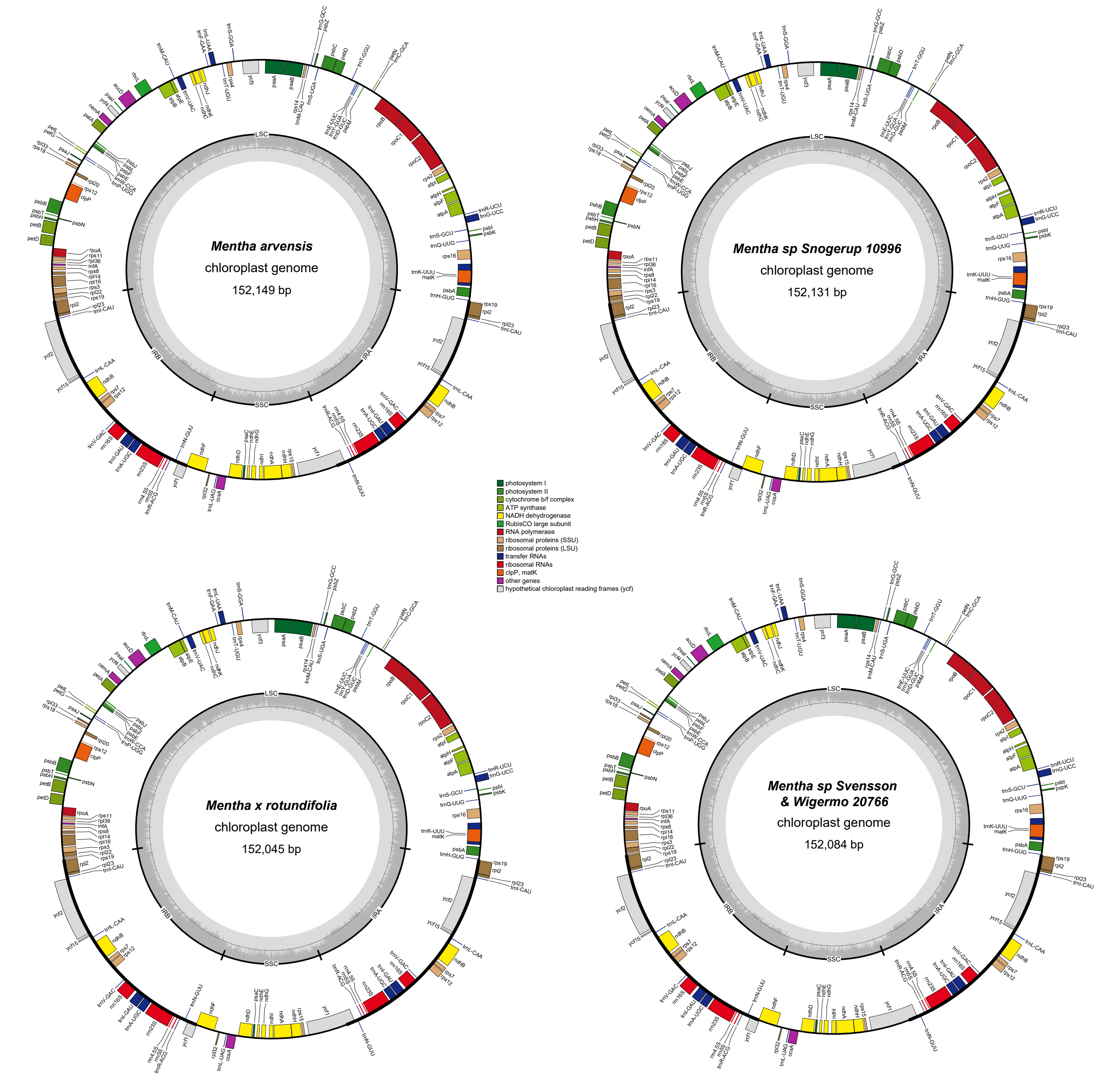
Mentha arvensis
chloroplast genome
152,149 bp
Mentha sp Snogerup 10996
chloroplast genome
152,131 bp
Mentha x rotundifolia
chloroplast genome
152,045 bp
Mentha sp Svensson & Wigermo 20766
chloroplast genome
152,084 bp
LSC
IRA
IRB
SSC
photosystem I
photosystem II
cytochrome b/f complex
ATP synthase
NADH dehydrogenase
RubisCO large subunit
RNA polymerase
ribosomal proteins (SSU)
ribosomal proteins (LSU)
transfer RNAs
ribosomal RNAs
clpP, matK
other genes
hypothetical chloroplast reading frames (ycf)
