# Supplementary material for: Comprehensive plastome variation and RNA editing in Mentha: insights into phylogenetic relationships and candidate DNA barcodes
Source: Front Plant Sci. 2026 Aug 18;17:1889271. doi: 10.3389/fpls.2026.1889271 (PMC13529761; doi:10.3389/fpls.2026.1889271)
Supplement: Supplementary file 2 [file DataSheet2.pdf]

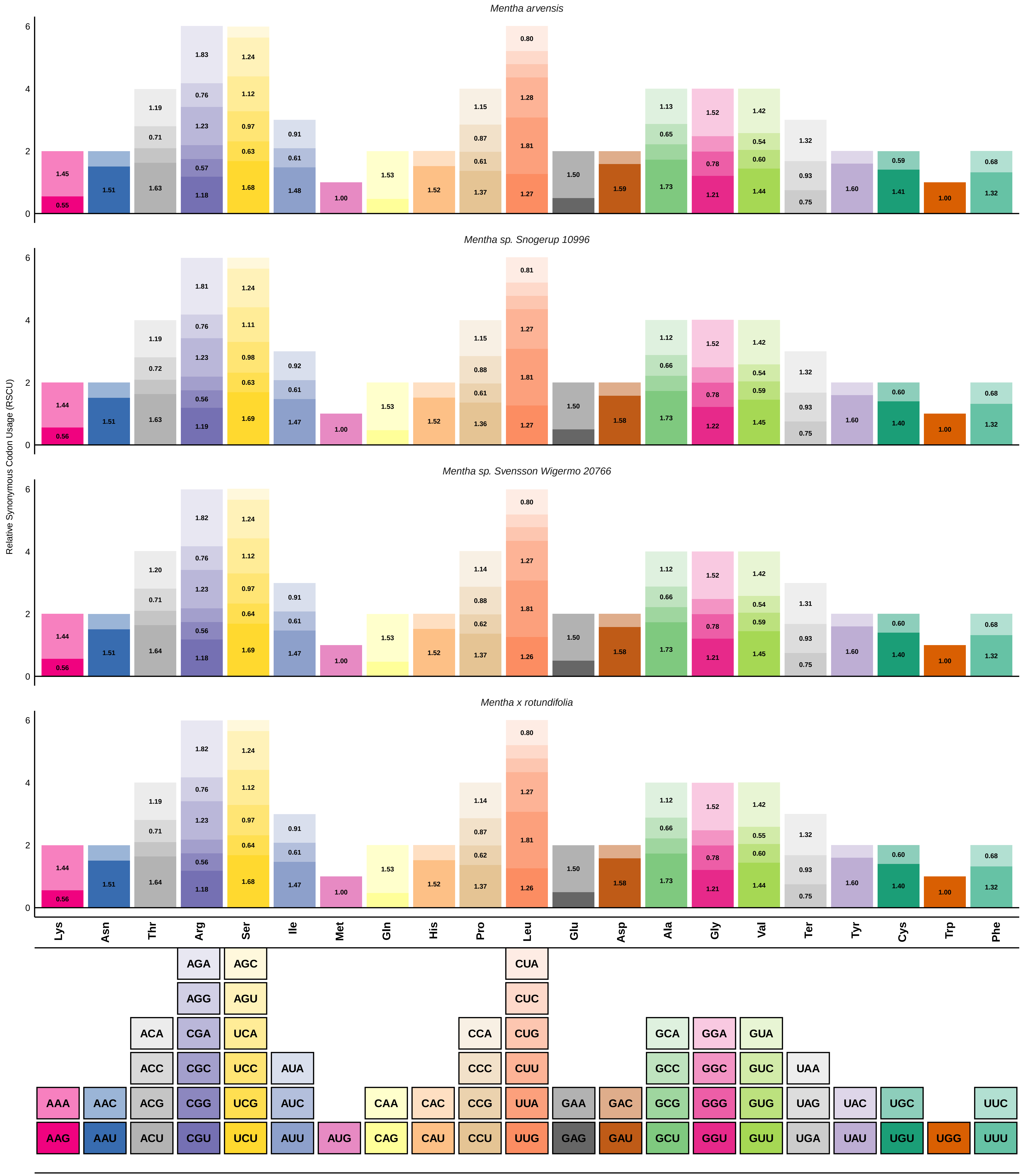
Relative Synonymous Codon Usage (RSCU)
0
2
4
6
Mentha arvensis
0.55 1.45 1.51 1.63 0.71 1.19 1.18 0.57 1.23 0.76 1.83 1.68 0.63 0.97 1.12 1.24 1.48 0.61 0.91 1.00 1.53 1.52 1.37 0.61 0.87 1.15 1.27 1.81 1.28 0.80 1.50 1.59 1.73 0.65 1.13 1.21 0.78 1.52 1.44 0.60 0.54 1.42 0.75 0.93 1.32 1.60 1.41 0.59 1.00 1.32 0.68
Mentha sp. Snogerup 10996
0.56 1.44 1.51 1.63 0.72 1.19 1.19 0.56 1.23 0.76 1.81 1.69 0.63 0.98 1.11 1.24 1.47 0.61 0.92 1.00 1.53 1.52 1.36 0.61 0.88 1.15 1.27 1.81 1.27 0.81 1.50 1.58 1.73 0.66 1.12 1.22 0.78 1.52 1.45 0.59 0.54 1.42 0.75 0.93 1.32 1.60 1.40 0.60 1.00 1.32 0.68
Mentha sp. Svensson Wigermo 20766
0.56 1.44 1.51 1.64 0.71 1.20 1.18 0.56 1.23 0.76 1.82 1.69 0.64 0.97 1.12 1.24 1.47 0.61 0.91 1.00 1.53 1.52 1.37 0.62 0.88 1.14 1.26 1.81 1.27 0.80 1.50 1.58 1.73 0.66 1.12 1.21 0.78 1.52 1.45 0.59 0.54 1.42 0.75 0.93 1.31 1.60 1.40 0.60 1.00 1.32 0.68
Mentha x rotundifolia
0.56 1.44 1.51 1.64 0.71 1.19 1.18 0.56 1.23 0.76 1.82 1.68 0.64 0.97 1.12 1.24 1.47 0.61 0.91 1.00 1.53 1.52 1.37 0.62 0.87 1.14 1.26 1.81 1.27 0.80 1.50 1.58 1.73 0.66 1.12 1.21 0.78 1.52 1.44 0.60 0.55 1.42 0.75 0.93 1.32 1.60 1.40 0.60 1.00 1.32 0.68
Lys Asn Thr Arg Ser Ile Met Gln His Pro Leu Glu Asp Ala Gly Val Ter Tyr Cys Trp Phe
AGA AGC CUA
AGG AGU CUC
ACA CGA UCA CCA CUG GCA GGA GUA
ACC CGC UCC AUA CCC CUU GCC GGC GUC UAA
AAA AAC ACG CGG UCG AUC CAA CAC CCG UUA GAA GAC GCG GGG GUG UAG UAC UGC UUC
AAG AAU ACU CGU UCU AUU AUG CAG CAU CCU UUG GAG GAU GCU GGU GUU UGA UAU UGU UGG UUU
